# Supplementary material for: Prognostic Significance of Prostate-Specific Antigen Persistence after Radical Prostatectomy: A Systematic Review and Meta-Analysis
Source: Cancers (Basel). 2021 Feb 24;13(5):948. doi: 10.3390/cancers13050948 (PMC7956516; doi:10.3390/cancers13050948)
Supplement: Supplementary file 1 [file cancers-13-00948-s001.pdf]

Table S1: Risk of Bias using the Quality In Prognosis Studies (QUIPS) tool

Author and year	Study Participation	Study Attrition	Prognostic Factor Measurement	Outcome Measurement	Study Confounding	Statistical Analysis and Reporting	Overall rating
Nasselli A 2008	low	low	low	low	moderate	moderate	*MODERATE*
Vesely S 2014	moderate	low	low	low	high/moderate	low	*MODERATE*
Bianchi L 2015	low	moderate	low	low	moderate	low	*MODERATE*
Fossati N 2017	low	low	low	low	moderate	low	*LOW*
Roy S 2018	low	moderate	low	low	moderate	moderate	*MODERATE*
Spratt DE 2018	low	moderate	low	low	low	low	*LOW*
Kim JK 2019	low	low	low	low	low	low	*LOW*
Preisser F 2019	low	low	low	moderate	low	low	*LOW*
Venclovas Z 2019	low	low	low	moderate	moderate	low	*MODERATE*
Overall rating per domain	*LOW*	*LOW*	*LOW*	*LOW*	*MODERATE*	*LOW*	
